# Hunting Metabolic Biomarkers for Exposure to Per- and Polyfluoroalkyl Substances: A Review

**DOI:** 10.3390/metabo14070392

**Published:** 2024-07-19

**Authors:** Xue Ma, Delei Cai, Qing Chen, Zhoujing Zhu, Shixin Zhang, Ziyu Wang, Zhengyan Hu, Haitao Shen, Zhen Meng

**Affiliations:** Zhejiang Provincial Center for Disease Control and Prevention, 3399 Binsheng Road, Hangzhou 310051, China

**Keywords:** per- and polyfluoroalkyl substances (PFAS), biomarker, metabolomics, toxicity

## Abstract

Per- and polyfluoroalkyl substances (PFAS) represent a class of persistent synthetic chemicals extensively utilized across industrial and consumer sectors, raising substantial environmental and human health concerns. Epidemiological investigations have robustly linked PFAS exposure to a spectrum of adverse health outcomes. Altered metabolites stand as promising biomarkers, offering insights into the identification of specific environmental pollutants and their deleterious impacts on human health. However, elucidating metabolic alterations attributable to PFAS exposure and their ensuing health effects has remained challenging. In light of this, this review aims to elucidate potential biomarkers of PFAS exposure by presenting a comprehensive overview of recent metabolomics-based studies exploring PFAS toxicity. Details of PFAS types, sources, and human exposure patterns are provided. Furthermore, insights into PFAS-induced liver toxicity, reproductive and developmental toxicity, cardiovascular toxicity, glucose homeostasis disruption, kidney toxicity, and carcinogenesis are synthesized. Additionally, a thorough examination of studies utilizing metabolomics to delineate PFAS exposure and toxicity biomarkers across blood, liver, and urine specimens is presented. This review endeavors to advance our understanding of PFAS biomarkers regarding exposure and associated toxicological effects.

## 1. Introduction

Per- and polyfluoroalkyl substances (PFASs) encompass both perfluorinated and partially fluorinated alkyl compounds, characterized by the replacement or partial replacement of hydrogen atoms by fluorine atoms on all carbon atoms. These synthetic chemicals consist of a carbon backbone ranging from 4 to 18 carbon atoms in length, terminating with functional groups [1]. Depending on the terminal functional groups, PFASs can be categorized into carboxylates, perfluorosulfonates, and phosphonates, among others. Furthermore, they can be classified based on carbon chain length, including perfluorooctanoic acid (PFOA), perfluorononanoic acid (PFNA), and perfluorodecanoic acid (PFDA). Owing to their exceptional industrial properties, such as hydrophobicity, oleophobicity, heat resistance, high stability, and low surface tension, PFASs have been extensively utilized since the 1950s in various industrial and consumer applications. These applications include the production of polytetrafluoroethylene, leather goods, waterproof textiles, firefighting foams, shampoos, cosmetics, and food packaging materials [2,3].

Unfortunately, the widespread use of PFASs and their desirable properties in commercial products have also led to their pervasive detection in the environment [4,5,6]. Currently, various concentrations of PFASs have been detected in environmental samples, animal serum, tissue samples, and human bodies worldwide, positioning PFASs as a new class of persistent organic pollutants, alongside polychlorinated biphenyls, organochlorine pesticides, and dioxins. Epidemiological and toxicological studies have shown that PFAS exposure results in toxic effects on the liver, nervous system, immune system, reproductive and developmental processes, genetics, and endocrine function, and may even induce tumors, although the metabolic toxicity remains unclear [7,8,9,10,11,12,13].

In recent years, representative PFASs such as perfluorooctane sulfonate (PFOS) have been listed under the Stockholm Convention [14]. Although these regulatory interventions have significantly reduced the emissions of PFOS and PFOA, human exposure to these compounds persists due to their persistence and ongoing production in some developing countries [15]. Moreover, the high-energy C-F covalent bond renders PFASs extremely resistant to degradation, leading to their persistence in the environment. They can contaminate water and food through direct discharge, migration, bioaccumulation, and biomagnification within marine ecosystems [16]. Evidently, even at low exposure levels, the potential health hazards of PFASs cannot be overlooked. As the adverse effects of PFASs on the environment and human health become more widely recognized, and as they migrate and biomagnify through the food chain, PFASs have emerged as a significant environmental and health concern.

Metabolomics, the large-scale study of metabolites within organisms, tissues, and cells, is considered a promising tool for elucidating the associations between environmental pollutants and health, as well as the etiology of certain diseases [17]. As an emerging technology, metabolomics characterizes small molecule metabolites present in cells and their roles in various biological processes, offering a novel and powerful approach to understanding the complex molecular responses induced by PFAS exposure. Despite extensive research on PFAS detection and toxicity, studies on the metabolic toxicity and biomarkers of PFASs remain limited. As metabolomics rapidly advances, reviewing the current state of research on PFAS metabolic toxicity and its biomarkers will provide timely guidance for future research in this crucial field.

This review briefly introduces the types of PFASs, sources of human exposure, and exposure characteristics. It summarizes the latest advancements in studying PFAS toxicity using metabolomics, reviews metabolic disturbances, potential exposure biomarkers, and effect biomarkers, and emphasizes metabolic toxicity and related metabolic biomarkers.

## 2. Introduction to PFAS

PFASs gained widespread application in consumer and industrial products during the 1990s due to their surfactant properties. Around the year 2000, the recognition of their potential toxicity led to the gradual phasing-out of legacy PFASs, primarily long-chain perfluoroalkyl carboxylic acids or perfluoroalkyl sulfonic acids. Currently, industrial applications of PFASs are increasingly shifting towards short-chain PFASs or those with chlorinated carbon or ether bond structures, giving rise to emerging PFASs.

### 2.1. Classification

PFASs are classified based on their functional groups and molecular structures into carboxylic acids and sulfonic acids. The carbon chain length for carboxylic acids generally ranges from 6 to 18, while for sulfonic acids it typically ranges from 5 to 10. Due to restrictions on the use of traditional long-chain PFAS, several emerging substitutes for conventional PFASs have gradually appeared. These substitutes include short-chain compounds and those incorporating fluorinated chlorinated carbons or ether bonds. Consequently, long-chain PFCA and PFSA are referred to as “legacy PFAS”, while the substitutes are termed “emerging PFAS”. Common types of PFASs are listed in Table 1.

### 2.2. Sources

The widespread presence of PFASs in industrial emissions, untreated domestic wastewater, sewage treatment plants, and aqueous film-forming foams has led to their detection in the environment, food, and human bodies [18,19,20,21]. The most significant pathways for human exposure to PFASs include food, drinking water, skin contact, indoor dust, and outdoor air, with food being the primary route [22,23]. Consuming contaminated food and drinking water, such as vegetables, crops, fish, meat, or processed food affected by PFAS, as well as drinking PFAS-contaminated water, can lead to exposure [19,24,25,26,27]. Additionally, contact with food packaging materials containing PFASs (e.g., food packaging paper and non-stick cookware) also poses a risk [28,29]. PFASs in soil may indirectly harm human health through bioaccumulation in crops [26].

PFASs can transfer into the human body via the food chain, accumulating and magnifying at each trophic level [30]. In plants, PFASs can be absorbed by roots from contaminated soil or water and then translocated to flowers, leaves, and fruits [31,32]. Long-chain PFASs tend to remain in roots, whereas short-chain PFASs are more readily transported to fruits [32,33]. Aquatic organisms ingest PFAS-contaminated sediments and seawater, with studies showing that short-chain PFASs easily accumulate in these organisms [34,35]. In livestock, animals ingest PFASs through water and feed, which then accumulate in muscle and milk [36,37], subsequently entering the human food chain and increasing human exposure risk.

### 2.3. Human Exposure Pathway and Characteristics

Multiple PFAS compounds have been detected in human tissues such as blood, kidneys, brain, cerebrospinal fluid, liver, lungs, and placenta [38,39,40,41,42]. This necessitates urgent research into PFAS exposure and its effects on these human samples.

#### 2.3.1. Population Characteristics

Occupational groups such as firefighters using aqueous film-forming foam have higher PFAS exposure risk than the general population, where diet is a critical factor influencing PFAS exposure [43]. High seafood consumption correlates with increased human PFAS levels [19]. PFAS serum levels exhibit gender differences, generally higher in males than females [44,45]. Menstruation and breastfeeding help reduce PFAS levels in females [46]. PFASs can also be transmitted to infants through umbilical cord blood, placenta, and breast milk [47,48]. Children are at higher risk of PFAS exposure than adults due to the presence of PFASs in formula milk and dairy products [27,49].

#### 2.3.2. Distribution Trend

With the gradual elimination of legacy PFASs, their levels in human serum are declining [50]. However, emerging PFASs can still be detected in serum, with some linked to adverse pregnancy outcomes and other toxic effects [51,52].

#### 2.3.3. Absorption and Distribution

PFAS absorption and distribution in organisms are influenced by carbon chain length, half-life, species, and gender. Animal studies show that perfluorohexanesulfonic acid (PFHxS), perfluorobutanesulfonic acid (PFBS), PFOA, and PFOS are almost completely absorbed after oral administration [53]. PFASs primarily enter the bloodstream through the intestinal barrier, binding to blood albumin and low-density lipoprotein, and then distribute to extra-intestinal organs [54]. Due to the high affinity for liver fatty acid-binding proteins, PFAS accumulation in the liver is well-documented [55]. PFASs can also undergo enterohepatic circulation, re-entering the intestines via bile and returning to the liver, leading to high liver concentrations [56]. Generally, longer carbon chains correspond to longer half-lives, ranging from hours to decades [57,58]. The half-life also varies by gender and species [59,60]. Current research mainly focuses on PFCA and PFSA, with the absorption, distribution, toxicokinetics, and metabolic toxicity of emerging PFASs requiring further investigation.

## 3. Toxic Effects of PFAS

Existing research has demonstrated that PFASs exhibit a range of toxic effects, including thyroid toxicity, hepatotoxicity, immunotoxicity, endocrine disruption, neurotoxicity, reproductive and developmental toxicity, and even carcinogenic and cancer-promoting properties. Additionally, elevated cholesterol levels, obesity, and endocrine disorders are associated with PFAS exposure. Although the connection between PFASs and toxicity in specific tissues and organs is recognized, the mechanisms through which PFASs influence metabolism and subsequently affect organ toxicity remain to be fully elucidated. This review summarizes the epidemiological and rodent model evidence linking PFAS-induced liver injury, reproductive and developmental toxicity, cardiovascular diseases, renal toxicity, and cancer, with a focus on metabolomics insights to better understand the metabolic mechanisms underlying PFAS exposure and toxic effects.

### 3.1. Liver Injury

Epidemiological studies have frequently associated PFASs, such as PFOA and PFOS, with elevated serum markers of liver injury (e.g., alanine aminotransferase (ALT), aspartate aminotransferase (AST), and alkaline phosphatase (ALP)), non-alcoholic fatty liver disease (NAFLD) grading, staging, and histopathological severity [61,62,63,64]. Rodent studies indicate that PFAS-induced hepatotoxicity manifests as liver enlargement, inflammation, steatosis, oxidative stress, apoptosis, and dysregulation of glucose and lipid metabolism [65,66,67,68].

Metabolomic studies have revealed that metabolic disturbances are critical in PFAS-induced liver damage. PFASs disrupt hepatic metabolism, particularly bile acid, amino acid, and lipid metabolism, leading to liver injury or influencing the development and susceptibility to NAFLD and liver damage [62,63,69,70]. Sen et al., through a correlation analysis of PFAS (PFOS, PFNA, PFOA) exposure levels with metabolic perturbations in the liver in 105 individuals with NAFLD, found that PFASs perturbed key metabolic pathways of bile acid metabolism and lipid metabolism in NAFLD, and that there were gender differences [63]. Similarly, it has been noted that high PFAS exposure levels were associated with more severe histology of liver damage in children with NAFLD, which may be related to elevated plasma levels of phosphoethanolamine, tyrosine, phenylalanine, aspartic acid, and creatine, and altered metabolites with reduced betaine levels [62]. Additionally, it has been found that prenatal PFAS exposure increases susceptibility to liver damage in childhood, associated with branched-chain amino acids, aromatic amino acids, and glycerophospholipid metabolism [69]. In addition, PFAS mixtures were found to be positively correlated with polipoprotein B (APOB) and γ-glutamyltransferase (GGT), and negatively correlated with direct bilirubin (DBIL) and total bilirubin (TBIL) [70].

Researchers analyzing metabolite changes in the liver or blood after PFAS exposure in rodents also show that PFASs affect bile acids, sterols, fatty acids, purine, and amino acid metabolism, contributing to liver injury [66,68,71,72,73]. For example, exposure to PFAS mixtures leads to liver injury in mice, such as increased liver weight, inflammation, and elevated plasma ALT levels, with significant changes in bile acid and sterol metabolism [71]. Lipidomic studies suggest that PFOS disrupts ceramide and lysophosphatidylcholine (LPC) metabolism, causing apoptosis and triglyceride depletion in liver cells, leading to morphological liver damage [72]. Functional characterization of lipid species showed that the fatty acid metabolic pattern of A/J mice was significantly altered by PFAS mixture exposure, especially the biosynthetic pathways of glycerophosphocholines (PC), glycerophosphoethanolamines (PE), and glycerophosphoserine (PS) in the liver were significantly affected [73]. In addition to bile acids, sterol, fatty acid, amino acid, and purine metabolism were also affected [66,68]. Jiang et al. examined liver nontargeted metabolomics after PFHxA exposure in mice and found that PFHxA substantially down-regulated xanthine and uric acid, increased GSH, and decreased GSSH, which presumably caused oxidative stress by interfering with purine metabolism and glutathione metabolism, thereby causing liver damage [66]. Additionally, PFASs may influence liver damage by affecting gut microbiota metabolism, as evidenced by altered fecal microbiota and arginine metabolism in PFOS-exposed mice [68].

### 3.2. Reproductive and Developmental Toxicity

Studies indicate that PFASs adversely affect male reproductive function, including semen quality and male reproductive hormones [74]. Similarly, PFASs impact female reproductive functions such as menstrual cycle regulation, hormone levels, and fertility [75]. Furthermore, PFASs can cross the placental barrier, thereby influencing fetal development and contributing to adverse pregnancy outcomes like preterm birth [76], reduced birth weight [77,78], postnatal growth [79], and neurodevelopmental disorders [80,81]. Additionally, PFAS exposure is linked to increased disease risks in offspring, such as liver damage susceptibility and type 1 diabetes [69,82]. Research has identified a correlation between maternal PFAS co-exposure and decreased sperm concentration, with varying contributions from different PFASs, with perfluoroheptanoic acid as a primary factor. However, no clear association was found between maternal PFAS exposure and testicular volume or reproductive hormones [74]. Contrarily, another study showed a significant negative correlation between PFAS mixtures and reproductive hormones, particularly estradiol (E2) and the E2/total testosterone (TT) ratio, with perfluoro-n-undecanoic acid (PFUdA) being a major contributor [83]. Higher serum concentrations of PFOA and PFHxS in pregnant women are associated with increased odds of preterm birth [76]. PFASs also adversely affect prenatal and postnatal neurodevelopment. Additionally, prenatal PFAS exposure is linked to neurodevelopmental disorders in children, such as reduced IQ performance and impaired executive function [80].

Population studies using metabolomics found that the effects of PFASs on preterm birth, fetal growth, childhood obesity trajectory, susceptibility to liver injury, and other reproductive developmental processes were related to amino acid, glycerophospholipid lipid and fatty acid, bile acid, uric acid, and carbohydrate metabolism [69,76,79,84,85]. Two studies found that PFAS exposure was associated with preterm delivery or fetal growth in pregnant women and that the effects were associated with metabolite alterations, e.g., one study found that the impact of prenatal PFAS exposure on the shortening of the gestation period was associated with eight metabolomic pathways and 52 metabolites in the neonatal blood spot [76,84]. Another study also found that higher concentrations of PFOA and PFNA in maternal serum were associated with reduced fetal growth and were strongly associated with disturbances in amino acid, lipid and fatty acid, bile acid, and androgen metabolism [84]. Two other studies found that prenatal exposure to PFASs was associated with increased susceptibility to liver damage and an obesity trajectory in children, as well as with metabolites such as amino acids, glycerophospholipids, sphingolipids, and octanoylcarnitine [69,79]. Consistent with human studies, laboratory research on rodents has demonstrated that the effects of PFASs on reproductive development were associated with metabolite disruption. For instance, pregnant Sprague–Dawley rats exposed to nafion byproduct 2 (NBP2), a type of perfluoroalkyl ether sulfonate, experienced neonatal mortality, reduced pup weight, and decreased liver glycogen in pups, as well as significant alterations in lipid and carbohydrate metabolism in dams and offspring [85].

### 3.3. Cardiovascular Toxicity

Cardiac metabolism, including conditions such as obesity, hypertension, and hyperlipidemia, has been extensively studied in relation to PFAS exposure. This review focuses on lipid and cholesterol abnormalities. Research indicates that twelve PFAS-related characteristic metabolites are associated with cardiovascular diseases [86]. A study examining the relationship between Cl-PFESA, a typical PFAS substitute in China, and the lipid profiles of 1336 residents in Guangzhou revealed that increased Cl-PFESA levels were positively associated with total cholesterol (TC), triglycerides (TG), low-density lipoprotein cholesterol (LDL-C), and high-density lipoprotein cholesterol (HDL-C) levels. The significant positive correlation between Cl-PFESA and lipid abnormalities suggests a detrimental effect of Cl-PFESA on lipid profiles, potentially exhibiting a nonlinear association [87].

Metabolomic studies have shown that PFASs primarily cause lipid and cholesterol abnormalities by disrupting bile acid and sterol metabolism [71,88,89,90]. Two population-based studies using metabolomics have found that PFASs were associated with plasma triglycerides and that this association was related to metabolite alterations [88,89]. Another study revealed that PFAS exposure was linked to a higher risk of dyslipidemia, and associations between PFASs and TC and LDL were found to be primarily related to glycerophospholipid metabolism, primary bile acid biosynthesis, and linoleic acid metabolism [90]. Animal studies have also demonstrated that PFASs interfere with metabolism, leading to increased circulating cholesterol and cholesterol metabolism abnormalities. For instance, C57BL/6J mice fed a high-fat diet and exposed to a PFAS mixture exhibited elevated fasting plasma cholesterol levels [71]. The levels of several sterol metabolites, including 4-cholesten-3-one, were upregulated, while most primary bile acids, such as chenodeoxycholic acid and β-muricholate, were significantly reduced in the liver metabolome. These findings suggest that PFASs may disrupt cholesterol metabolism and transport, contributing to a PFAS-induced cholesterol imbalance.

### 3.4. Glucose Homeostasis Disruption

PFASs are closely associated with endocrine disorders such as diabetes, impaired glucose tolerance, overweightness, or obesity [91]. Epidemiologic studies have found that PFOA and PFOS exposure were associated with higher 30 min blood glucose levels and area under the glucose curves (AUCs) during oral glucose tolerance tests (OGTTs), as well as higher levels of HbA1c [89,92]. Additionally, research indicates that PFOA and PFHxS were associated with increased 2 h glucose levels and increased area under the glucose curve, as well as altered lipid (e.g., sphingolipids, linoleic acid, and de novo lipogenesis) and amino acid (e.g., aspartic acid and aspartate, tyrosine, arginine, and proline) metabolism [93]. Laboratory evidence further suggests that gastric gavage of pregnant rats with low doses of PFOSs disrupts critical metabolic products, such as glycerol-3-phosphate and lactosylceramide, affecting fasting blood glucose (FBG) in mothers and thus impacting glucose homeostasis [94].

### 3.5. Other Toxicological Impact

Studies have demonstrated that PFASs can also interfere with metabolism and impact the development of kidney diseases and cancer [95,96,97]. Research indicates a causal relationship between PFASs and declining kidney function and chronic kidney disease [95]. A study identified eight potential biomarkers associated with PFAS exposure, all significantly correlated with kidney function markers, suggesting adverse effects of high PFAS exposure on kidney function [96]. Furthermore, PFASs have been implicated in liver cancer risk, possibly through metabolic interference. For instance, plasma PFAS concentrations are positively correlated with hepatocellular carcinoma (HCC) risk, with PFOSs showing the strongest correlation, associated with increased HCC incidence [97]. Metabolomic analysis revealed 433 metabolites in plasma associated with PFOSs and 499 metabolites associated with HCC.

## 4. Potential Biomarkers

The metabolome encompasses the complete set of small molecules that participate in physiological processes within the body or in cells and tissues. The application of high-throughput analytical techniques has emerged as a novel tool for the comprehensive examination of endogenous metabolites in vivo. Research has demonstrated that pollutants can disrupt metabolic pathways, leading to alterations in metabolite levels within the human body. These changes in metabolites may indicate defects in specific pathways or the activation of certain signals, and the dynamic alterations of these metabolites can serve as biomarkers of pollutant-induced damage to the human body. Metabolomics can reveal toxicological changes and related mechanisms at an earlier stage and can be utilized as a tool for discovering biomarkers of pollutant exposure and effects. Metabolomics primarily focuses on the quantitative analysis of metabolites under specific physiological conditions following organismal exposure, understanding metabolic changes under varying conditions, and elucidating the associations between these changes and the organism’s health status and disease. Therefore, comprehending the relationship between PFAS exposure and metabolites within the body can provide additional evidence for elucidating the health risks posed by pollutant exposure.

### 4.1. Exposure Biomarkers

Metabolomics methodologies emphasize the analysis of primary and secondary metabolites, which are crucial for obtaining the phenotypic fingerprints of organisms in their environments. These metabolic gatekeepers can also elucidate molecular connections between exposure biomarkers and health outcomes, which might otherwise be obscured by the complex interactions present in direct measurements [98]. By focusing on the quantitative analysis of metabolites in organisms exposed to PFASs under specific physiological conditions, metabolomics can identify metabolic changes induced by PFASs and their associations with health status. Consequently, we concentrate on the quantitative analysis of metabolites in blood, liver, and urine under specific physiological states of PFAS exposure, summarizing the disturbances in metabolites in both human populations and rodent models post-PFAS exposure to identify potential PFAS-related exposure biomarkers.

Current research on PFAS exposure and metabolic alterations primarily involves human and rodent subjects. The studies cover various PFAS compounds, with the most extensive research on PFOA, PFOS, PFHxS, PFNA, and PFDA. There are also some metabolomics studies on emerging PFASs, such as 6:2 FST, 6:2 Cl-PFESA, GenX, NBP2, and FTEOs, though most research focuses on the effects of individual PFAS compounds, with some addressing the impact of mixtures. In the study of PFAS-related metabolites, alterations in amino acids (with branched-chain amino acids being more prominently affected), lipids, bile acid metabolism, and the urea cycle are common metabolomic features. Glycerophospholipid metabolism in lipid pathways is considered a key metabolic feature, along with fatty acid and carnitine metabolism related to fatty acid oxidation and energy supply pathways. Purine and pyrimidine metabolism in cellular energy systems are also identified as significant metabolic changes, with potential as future exposure biomarkers [99].

Most current studies utilize metabolomics in the blood (serum, plasma, cord blood) and liver to identify related metabolites, with fewer studies on urine and placental metabolite changes. Some studies simultaneously use serum or plasma and liver samples to explore the impact on enterohepatic circulation. Given the greater practicality of detecting metabolites in different samples for clinical translation, we summarize recent studies and advancements in metabolomics from various samples, as detailed in Table 2 and Table 3.

#### 4.1.1. Blood

As illustrated in Table 2 and Table 3, in recent years there have been 19 studies investigating the effects of PFASs on metabolomic alterations in blood (plasma, serum, or cord blood). In human studies, PFAS exposure detection and metabolomic analyses typically utilize the same sample matrix, encompassing the general population, special populations (pregnant women, fetuses, children), occupational groups, and diseased cohorts. Diseased cohorts primarily include overweight or obese children, adolescents or young adults, adults and children with NAFLD, HCC cases, and individuals at high risk for or diagnosed with type 2 diabetes. Additionally, a few studies have inconsistent samples for PFAS exposure detection and metabolomic analyses, such as examining maternal PFAS exposure and subsequent metabolomic changes in cord blood, newborn, or child blood to investigate prenatal exposure effects on offspring blood metabolomics.

PFAS-related studies using metabolomics have found that PFAS exposure is primarily associated with altered metabolism of amino acids, lipids, glycerophospholipids, fatty acids, and bile acids in blood [69,76,90,100,101,102]. For example, PFAS exposure has been found to be primarily associated with altered lipid metabolism in the blood, including phospholipid metabolism, long-chain saturated fatty acids, fatty acid metabolism (acylcarnitines, monounsaturated fatty acids), medium-chain fatty acids, and long-chain polyunsaturated fatty acids (n3 and n6), in studies of the male population, pregnant women and in the elderly population [90,100,101]. Two population studies found that increased PFAS exposure was associated with serum amino acids, such as valine, leucine, isoleucine, tryptophan, and alphaketobutyrate, as well as glycerophospholipid phospholipids (PCs) aa C36:1, 1-palmitoyl-GPC (16:0), and Lyso-PC a C18:1 by examining the metabolomics correlation analyses of PFAS exposure [69,101]. In the elderly population, Yang et al. found that the nine metabolites most strongly associated with PFASs mainly included four fatty acyl groups, such as oleoyl-L-carnitine, 7α-hydroxy-3-oxo-4-cholestenoic acid (7-HOCA), 22β-hydroxycholesterol and vitamin D3, three steroids and steroid derivatives, lipids, and lipid-like substances including γ-tocopherol and PG (16:0/18:1) [100]. In addition, two population studies have found that PFAS exposure was primarily associated with the metabolism of lipids and bile acids, among others, involving polyunsaturated omega-6 fatty acids, arachidonic and linoleic acids, cholesteryl esters, LPE, alkylPCs, stearic acid, arachidic acid, monounsaturated palmitoleic acid, linoleic acid, HCA, TaMCA, GCDCA, CA, and many other metabolites [76,102]. Some studies have also found that PFASs were associated with disturbances in purine–pyrimidine metabolism, and retinol and sterol metabolism [51,103]. For example, PFAS exposure in pregnant women predominantly affects fatty acid and retinol metabolism [51]. In children, PFAS exposure is associated with pathways involved in de novo fatty acid biosynthesis, the tricarboxylic acid (TCA) cycle, and pyrimidine and purine metabolism [103].

**Table 2:** PFAS exposure and associated metabolites in epidemiological studies.

PFAS	Study Object	Sample Size	Sample Matrix	Main Finding(s)	Ref.
PFOA, PFNA, PFDA, PFUdA, PFTrDA, PFHxS, PFOS, 6:2 FTS, etc.	Non-occupationally exposed residents near industrial parks of Shandong Province	91	serum	PFOA and PFOS were associated with 126 and 117 metabolites, respectively The most highly correlated metabolites were predominantly lipids and lipoids, including oleoyl-L-carnitine, 5,6-dihydroxy-8Z,11Z,14Z-eicosatrienoic acid (5,6-DHET), dec-anoyl-L-carnitine, 9,10- dihydroxy-12-octadecenoic acid (9,10-DHOME), 7α-hydroxy-3-oxo-4-cholestenoic acid (7-HOCA), 22β-hydroxycholesterol, vitamin D3, γ-tocopherol, and PG (16:0/18:1)	Yang Y et al., 2024 [100]
PFOS, PFHxS, PFOA, PFNA	Pregnant African American Newborns in Atlanta, Georgia	267	serum	435, 559, 154, and 1262 metabolites were associated with PFOA, PFOS, PFNA, and PFHxS, respectively Contains carnitine and bile acids, polyunsaturated omega-6 fatty acids, arachidonic, linoleic acids, indole-3-acetic acid methyl ester, 4-hydroxy-3-methyloxybenzeneglycol, β-nicotinamide adenine dinucleotide, benzoic acid, s -lactic acid, and glutathione, etc.	Taibl KR et al., 2023 [76]
23PFSA, mainly PFHpA, PFOA, PFBS, PFPeS, PFHxS, PFHpS, PFOS, 6:2 FTS	Occupational workers and residents of fluorine chemical plants in Hubei, China	225	urine	78 metabolites associated with PFAS exposure	He A et al., 2023 [96]
PFOS, PFHxS, PFHpS, PFOA, PFNA, PFDA	The Study of Latino Adolescents at Risk (SOLAR), Southern California Children’s Health Study (CHS)	312, 137	plasma	463 and 200 metabolites of amino acids, lipids, prostaglandin formation from arachidonate and de novo fatty acid biosynthesis, and the aromatic amino acid metabolic pathway associated with PFASs in the SOLAR and CHS cohorts, respectively Thyroxine (T4), L-glutamic acid, equine uric acid, arachidonic acid, and aminoadipic acid were positively associated with PFAS Triglycerides and acetoacetate were negatively associated with PFASs in the SOLAR cohort but positively associated with PFASs in the CHS cohort	Goodrich JA et al., 2023 [104]
PFOA, PFOS, PFHXS, PFDA, PFUdA, PFNA	Cohort investigating pregnant women and children in the US VDAART cohort study	459, 401	plasma	43 metabolites of sphingomyelin, lysophospholipid(LPL), long chain polyunsaturated fatty acids were associated with PFASs in the parent, containing 17 amino acid metabolites 80 metabolites of LPL and phosphatidylcholine (PC) metabolic pathways were associated with PFASs in child, containing 20 amino acid metabolites The most associations with PFASs were LPL and PC pathway metabolites, with the smallest *p* value being 1-palmitoyl-GPC (16:0)	Prince N et al., 2023 [101]
PFHxS, PFOA, PFHpS, PFNA, PFOS, 6:2 Cl-PFESA, PFDA, PFUdA, 8:2 Cl—PFESA	Male residents recruited from Guangzhou	278	serum	51, 137, 34, 112, 153, 112, 101, 49, and 46 metabolites related to PFHxS, PFOA, PFHpS, PFNA, PFOS, PFDA, PFUdA, 6:2Cl-PFESA, and 8:2Cl-PFESA, respectively Glycocholic acid was negatively correlated with PFOS; bile acids were negatively correlated with PFNA, PFOS, PFDA, and PFUdA; deoxycholic acid was negatively correlated with PFOS, PFDA, PFDA, and 8:2 Cl- PFESA glycerophospholipids, aryl alcohol lipids, and fatty acyls metabolites related to Cl-PFESA	Chen Y et al., 2023 [90]
PFHxS, PFNA, PFOA, 2 PFOS isomers	Cohort with NAFLD who underwent laparoscopic bariatric surgery	105	Liver, serum	PFASs were correlated with hepatic primary BAs (TCA, GCDCA, TCDCA) and hepatic secondary BAs (DCA, GHCA and GUDCA) PFOA and PFOS were positively correlated with Cers (e.g., Cer(d18:0/16:0), HexCer(d18:1/18:0)), ether phospholipids (e.g., O-PC(40:4)), TGs, and DGs	Sen P et al., 2022 [63]
PFOS, PFHxS, PFOA, PFDA, PFNA, PFUdA	A nested case-control study of HCC	50 pairs	plasma	433 metabolites associated with high levels of PFOS exposure	Goodrich JA et al., 2022 [97]
PFOA, PFNA, PFDA, PFUdA, PFHxS, PFHpS, 6:2 Cl-PFESA, 8:2 Cl-PFESA	Matched samples of pregnant women delivering in Beijing’s hospitals	84 pairs	serum, cord blood	279 and 338 metabolites were associated with PFASs in maternal and cord serum, respectively PFOA and PFHxS were the two PFASs with high correlation to metabolites, with 190, 218(maternal) and 201, 215 (cord blood) metabolites, respectively Two Cl-PFESAs were less relevant to metabolites, both having only one in the maternal blood	Li Y et al., 2021 [51]
PFOS, PFOA, PFHxS, PFNA, EtFOSAA, MeFOSAA, PFDA, PFOSA	A Diabetes Prevention Program project for a multicenter randomized clinical trial in people at risk for type 2 diabetes	691	plasma	17, 17, 10, 30, 23, and 34 metabolites were associated with total PFOS, n-PFOS, Sm-PFOS, total PFOA, n-PFOA, and Sb-PFOA, respectively Sphingolipids and glycerophospholipids were positively correlated with PFAS, including C34:2 PE, C36:2 PE, C34:1 PC and C36:1 PC, etc. PFOA and PFOS were correlated with phosphatidylethanolamine, triacylglycerols, C16:1 SM, and C18:2 SM PFASs correlated with branched-chain amino acids (isoleucine, leucine, valine) and glycine	Mitro SD et al., 2021 [105]
PFHxS, PFOS, PFOA, 6:2 Cl-PFESA, PFNA, PFDA, PFUdA, PFHpS	Cord blood samples stored in the Beijing Cord Blood Bank	104	cord blood	73 metabolites were associated with PFAS, including lipids, free fatty acids, and bile acids Mainly correlated with cholesteryl esters, LPE, alkylPCs, stearic acid, arachidic acid, monounsaturated palmitoleic acid, linoleic acid, HCA, TaMCA, GCDCA, and CA	Sinisalu L et al., 2021 [102]
PFOA, PFOA, PFHxS, PFDA, PFNA, PFUdA	A case-control study on T2D	187 pairs	plasma	171 metabolites were associated with PFAS 19, 14, 18, 66, 62, 59 metabolites related to PFOS, PFOA, PFHxS, PFDA, PFNA and PFUdA, respectively	Schillemans T et al., 2020 [91]
PFOS, PFOA, PFNA, PFHxS, PFUdA	The Human Early-Life Exposome project	1105 pairs	serum	66 and 34 metabolites were associated with PFASs at high and low risk of liver injury, respectively. Including amino acids, biogenic amine, glycerophospholipids, sphingolipids, and hexose	Stratakis N et al., 2020 [69]
PFOA, PFOS and PFHxS	NAFLD patients enrolled at Children’s Healthcare of Atlanta	74	plasma	348, 349, and 662 metabolites were associated with PFOA, PFOS, and PFHxS, respectively	Jin R et al., 2020 [62]
PFOA, PFOS, PFHxS	Overweight and Obese Hispanic Children in Downtown Los Angeles	40	plasma	149, 298, and 17 metabolites were associated with PFOA, PFOS, and PFHxS, respectively	Alderete TL et al., 2019 [93]
PFOA, PFOS, PFNA, PFHxS	The Health Outcomes and Measures of the Environment Study	114	serum	Negative ion pattern, 17, 63, 47, and 29 metabolites associated with PFOA, PFOS, PFNA, and PFHxS, respectively Positive ion mode, 18, 253, 76, and 39 metabolites associated with PFOA, PFOS, PFNA, and PFHxS, respectively	Kingsley SL et al., 2019 [103]

PFOA, perfluorooctanoic acid; PFNA, perfluorononanoic acid; PFDA, perfluorodecanoic acid; PFTrDA, perfluorotridecanoic acid; PFHxS, perfluorohexane sulfonic acid; PFOS, perfluorooctane sulfonic acid; 6:2 FTS, 6:2 fluorotelomer sulfonic acid; PFHpA, perfluoroheptanoic acid; PFBS, perfluorobutane sulfonic acid; PFPeS, perfluoropentanc sulfonic acid; PFHpS, perfluoroheptane sulfonic acid; PFUdA, perfluoroundecanoic acid; 6:2 Cl-PFESA, 6:2 chlorinated polyfluorinated ether sulfonate; 8:2 Cl-PFESA, 8:2 chlorinated polyfluorinated ether sulfonate; EtFOSAA, 2-(N-ethyl-perfluorooctane sulfonamido) acetic acid; MeFOSAA, 2-(N-methyl-perfluorooctane sulfonamido) acetic acid; PFOSA, perfluorooctane sulfonamide.

**Table 3:** PFAS exposure and associated metabolites in rodent studies.

PFAS	Study Object	Dose	Sample Matrix	Main Finding(s)	Ref.
PFOA	Humanized PPARα mice	8 μM/kg, 6–7 weeks	liver	Positively correlated with Cer(18:0/23:0), LPE, DG(36:2), TG(18:1/18:1/18:1), TG(50:3), and TG(51:3), and negatively correlated with HexCer(18:1/22:0) and SM(d18:0/24:1)	Sen P et al., 2022 [63]
HFPO-DA or “GenX”, NBP2	C57BL/6 mice	0.5, 5, 100 mg/kg, 28 d	liver	199 and 123 metabolites associated with GenX and NBP2 group, respectively, and 54 and 86 common differential metabolites in males and females, respectively The most common dysregulated fatty acids were oleic acid (OA) and dihomo-γ-linoleic acid (DGLA)	Kirkwood-Donelson KI et al., 2024 [106]
FTEOs, PFOA	CD-1 mice	5, 100 ng/L, 17.5 d	placenta	14 related metabolites including asparagine, fatty acids, glucose, threonine, lactate, lysine, and threonine PFOA increased glucose and threonine and decreased creatine. FTEOs decreased asparagine and lysine and increased creatine	Adams H et al., 2024 [107]
PFOS	SD rats	0.03, 0.3 mg/kg, 18 d	liver	Negative ion mode, 164 and 158 metabolites in low and high dose groups, respectively, including E, 5′-Hydroxy-3′,4′,7-trimethoxyflavan, tragopogonsaponin F, enkephalin L, and tetracosapentaenoic acid (24:5n-3), etc. Positive ion model, 104 and 56 metabolites in low- and high-dose groups, respectively; the major metabolites were glycerophospholipids, carboxylic acids and their derivatives, fatty acyls, and aryl alcohol lipids	Yu G et al., 2023 [94]
Mixture of PFOA, PFNA, PFDA, PFUdA, PFDoDA, PFTrDA, PFTeDA, PFOS	A/J mice	3 g/week, 10 weeks	liver	95 and 72 differential metabolites in male and female mice, respectively, and 54 metabolites were present in both sexes PC 38:4, PC 38:5, PC 38:6, TG 52:5, TG 56:5, and PE 38:6; PC 34:2 differed in both sexes; TG 54:3 and TG 56:6 differed in females; PC 36:4 and TG 56:8 differed in males 4 fatty acids, alpha-linolenic acid, DHA, DPA, and FA 20:4, were significantly different in both sexes	Khan EA et al., 2023 [73]
PFOA	C57BL/6 mice	1 ppm/kg, 40 ppb/kg, 4 weeks	Serum, liver	In serum, 145 and 83 differential metabolites in high and low dose group, respectively, including TAG 62:13 and TAG 62:14, PC (32:2), PC (33:2), PC (35:4), LPC 20:3, and PI 36:4, etc. In liver, 268 and 117 differential metabolites in the high- and low-dose group, respectively. PFOA increased unsaturated triglycerides and decreased sphingomyelin, saturated phosphatidylcholine, saturated lysophosphatidylcholine, and phospholipids ethers	Gao B et al., 2022 [108]
Mixture of POPs with six PFAS: PFHxS, PFOS, PFOA, PFNA, PFDA, PFUdA	NOD/SHiLtJ mice	0.14, 2.9 μg/kg, 12 weeks	serum	Bile acid dysregulation, TDCA, and GDCA were negatively correlated with PFAS, LCA was positively correlated with PFAS, and most BAs were negatively correlated LPC 18:2, LPC 20:4, LPC 22:5, and LPC 22:6 were negatively correlated with PFAS, whereas LPC 20:3 was positively correlated with PFAS; 48 PCs were correlated with PFAS 3 and 19 polarity-differentiated metabolites in the low- and high-dose groups, respectively, and PFASs were negatively correlated with serine, threonine, glycerol-3-phosphate, palmitic acid, linoleic acid, and cholesterol; it was positively correlated with 3-hydroxybutyrate, fumaric acid, glutamate, and uric acid	Sinioja T et al., 2022 [82]
PFAS mixture, PFOA, PFOS, PFNA, PFHxS, GenX	C57BL/6J mice	10 mg/L, 12 weeks	plasma, liver	PFAS exposure altered sterol, bile acid, ketone body, and acylcarnitine metabolites in the liver and circulatory system with inconsistent alterations The hepatic sterol metabolites cholesterol sulfate and 7-HOCA were decreased, and 4-cholesten-3-one was increased; ketone body 3-BHBA was increased; most of the acylcarnitines were increased Plasma levels of the sterol metabolites cholesterol and 7-HOCA were increased; metabolites of bile acids cholate, chenodeoxycholate, and deoxycholate were increased; ketone bodies 3-BHBA were increased; acylcarnitines C6 and C8 were decreased	Roth K et al., 2021 [71]
PFHxA	ICR mice	50, 200 mg/kg, 2 months	Serum, liver	30 and 49 differential metabolites in serum and liver, respectively, mainly interfering with purine and glutathione metabolism Xanthine and uric acid in liver decreased. In serum, xanthine increased and uric acid level increased slightly with the increase in PFHxA	Jiang L et al., 2021 [66]
PFOS	BALB/c mouse	100, 1000 μg/kg, 2 months	liver	54 differential lipid metabolites, mainly glycerophospholipids, sphingolipids, and TGs Eight PCs, eight LPCs, two ceramides, four phosphatidylinositols (PIs), four lysophosphatidylinositols (LPIs), and one lysophosphatidylethanolamine (LPE) were up-regulated, while nine TGs, eight sphingomyelins (SMs), three cardiolipins (CLs), two phosphatidylethanolamines (PEs), three phosphatidylglycerol (PG), and two coenzymes were downregulated.	Li X et al., 2021 [72]

HFPO-DA or “GenX”, hexafluoropropylene oxide dimer acid; NBP2, nafion byproduct 2; 6:2 FTEOs, 6:2 fluorotelomer ethoxylates; PFDoDA, perflurododecanoic acid; PFTeDA, perfluorotetradecanoic acid; PFHxA, perfluorohexanoic acid.

The close association of PFAS exposure with the metabolism of lipids, amino acids, and bile acids is also well documented in metabolome-related studies in disease populations [62,93,97,105]. In studies of PFAS-related metabolites examined in children with NAFLD, in populations at high risk for diabetes, and in overweight and obese populations, it was found that amino acid and glycerophospholipid metabolisms were most relevant after PFAS exposure [62,93,105]. For example, the pathways found to be most affected after PFAS exposure in children with NAFLD include tyrosine metabolism, alanine and aspartate metabolism, glycine, serine, alanine and threonine metabolism, urea cycle metabolism, and glycerophospholipid metabolism [62]. Differential metabolites have been found in overweight and obese children, mainly associated with amino acids such as aspartate, tyrosine, arginine, and proline and lipid metabolic pathways such as sphingolipid metabolism, fatty acid metabolism, de novo lipogenesis, and linoleic acid metabolism [93]. In people at high risk of diabetes, PFASs are mainly associated with amino acid and glycerophospholipid metabolism, and PFASs are closely related to branched-chain amino acids (isoleucine, leucine, and valine) [105]. In addition, metabolites have been found to be closely associated with PFOS in HCC cases, mainly interfering with amino acids, carbohydrates, and glycan biosynthesis, and metabolism in relation to them [97].

Similarly, several animal studies utilizing blood samples have also analyzed blood metabolomics by PFAS exposure and found that PFAS exposure is also primarily associated with lipid and bile acid metabolites [66,71,82,108]. A comprehensive metabolomics study examining lipid, bile acid, and sterol metabolites found that PFAS exposure in both sexes increased plasma levels of sterol metabolites, bile acids, and ketone bodies, while acylcarnitine levels decreased [71]. Among them, bile acids, such as cholate (CA), chenodeoxycholate (CDCA), beta-muricholate, deoxycholate (DCA), taurodeoxycholate (TDCA), ursodeoxycholate (UDCA), tauroursodeoxycholate (TUDCA), and hyocholate, significantly increased, and cholesterol, 7-alpha-hydroxy-3-oxo-4-cholestenoate (7-HOCA), and 4-cholesten-3-one levels increased among sterol metabolites. Additionally, plasma levels of acylcarnitines, such as hexanoylcarnitine (C6) and octanoylcarnitine (C8), decreased. Similarly, PFAS exposure during pregnancy also dysregulated bile acid and lipid metabolism in mice, with taurine deoxycholic acid (TDCA) and glycodeoxycholic acid (GDCA) negatively correlating with PFAS, lithocholic acid (LCA) positively correlating with PFAS, and the majority of the BAs trending towards a negative correlation with PFASs [82]. In addition, a serum lipid metabolome of male mice found that PFOA altered 11 lipids, including PC and PI, at either high or low doses compared to controls [108]. Two other studies found that PFAS exposure also interfered with amino acid, fatty acid, purine, and glutathione metabolic pathways in the blood, with PFASs negatively correlating primarily with serine, threonine, aspartic acid, glycerol-3-phosphate, palmitic acid, linoleic acid, stearic acid, arachidonic acid, cholesterol, and 1,5-nonhydrohexanol [66,82]. PFASs positively correlated with 3-hydroxybutyric acid, fumaric acid, methionine, glutamic acid and gluconic acids, uric acid, and glycerol monostearate [82].

#### 4.1.2. Liver

The impact of PFAS exposure on liver metabolites primarily manifests in disruptions to bile acid and lipid metabolism, as well as effects on amino acid, purine, and glutathione metabolism. Human studies on liver metabolomics are limited, likely due to the difficulty in obtaining liver samples. However, one study involving liver biopsies from individuals with NAFLD undergoing laparoscopic surgery analyzed the relationship between PFAS exposure levels in blood and liver metabolites, finding that PFAS exposure was primarily associated with disruptions in liver bile acid and lipid metabolic pathways [63]. In individuals with NAFLD, studies have demonstrated gender-specific associations between PFAS exposure and liver metabolites. In females, serum concentrations of PFASs (PFNA, PFOA, and PFOS) positively correlated with primary liver bile acids (TCA, GCDCA, TCDCA), while PFOA was positively associated with various secondary bile acids (DCA, GHCA, and GUDCA). Additionally, PFOA and PFOS were positively correlated with ceramides (e.g., Cer(d18:0/16:0), HexCer(d18:1/18:0)), ether phospholipids (e.g., O-PC(40:4)), TGs), and diglycerides (DGs). These associations were not observed in males. For both sexes, shared metabolic impacts included overexpression of primary bile acid biosynthesis, glycerophospholipid metabolism, and alanine, aspartate, and glutamate metabolism. Similar lipid associations were observed in mice, where PFOA exposure was linked to Cer, hexosylceramides(HexCer), LPE, DG, and TG. Most studies linking PFAS exposure and liver metabolomics focus on rodent models, particularly targeting lipid metabolism. Three animal studies have all found that PFAS exposure leads to alterations in the hepatic metabolome and is primarily associated with lipid metabolism [72,73,106]. For example, Kirkwood-Donelson KI et al. studied the effects of NBP2 or GenX exposure on the hepatic metabolome and found that over half of the detected lipids were significantly dysregulated, with oleic acid (OA) and dihomo-γ-linoleic acid (DGLA) being the most commonly affected fatty acids [106]. Khan et al. found that, similar to the blood metabolome, lipid differential metabolites were dominated by eight different lipid major classes, including Cer, DG, PC, PE, PS, SM, sterols, and TG, with common lipid species across genders including PC 38:4, PC 38:5, PC 38:6, TG 52:5, TG 56:5, and PE 38:6 [73]. In addition, further studies on fatty acids revealed that four fatty acids, alpha-linolenic acid, DHA, DPA, and FA 20:4, were significantly different between males and females. Li et al. analyzed PFAS exposure and lipid metabolites and found that 54 lipids had differentiated lipid metabolites and that the major abnormal lipids were glycerophospholipids, sphingolipids, and TG [72]. In addition, in studies analyzing the metabolism of sterols, ketone bodies, and acylcarnitines in the liver, it was found that sterol metabolites in the liver mainly showed a decrease, ketone bodies an increase, and most of the acylcarnitines an increase [71]. In addition, two animal studies have found that PFAS exposure is associated with amino acid, purine, and glutathione metabolism [66,94]. Among them, Yu et al. found that more metabolites were found in the negative ion pattern and more differential metabolites were up-regulated after PFAS exposure. These metabolites were enriched in pathways such as α-linolenic acid metabolism, linoleic acid metabolism, purine metabolism, glycine, serine, and threonine metabolism, steroid biosynthesis, glycolysis/gluconeogenesis, and glucagon signaling pathways [94]. Jiang et al. found that PFHxA exposure was also associated with purine and glutathione metabolism [66].

#### 4.1.3. Urine and Placenta

Limited research has examined metabolite changes in urine and placenta. He et al. found that PFAS concentrations in urine closely correlated with those in serum among workers and residents near a large fluorochemical plant in Hubei Province, China, suggesting that urinary PFAS could serve as a good indicator of serum PFAS levels [96]. Eight potential exposure biomarkers were identified, with differential metabolites associated with amino acids, steroids, fatty acid derivatives, carboxylic acids, isoprenoids, amines, and vitamin pathways. Adams et al. investigated placental metabolites in healthy CD-1 pregnant mice exposed to PFOA and FTEOs, identifying significant changes primarily in amino acids [107]. Fourteen different metabolites, including asparagine, fatty acids, glucose, threonine, lactate, lysine, and creatine, were identified. PFOA exposure led to increased glucose and threonine and decreased creatine in the placenta, whereas FTEO exposure decreased asparagine and lysine while increasing creatine. Pathway analysis indicated alterations in glycine, serine, and threonine metabolism, as well as valine, leucine, and isoleucine biosynthesis following PFOA exposure, with biotin metabolism affected by FTEO exposure.

### 4.2. Effect Biomarkers

There is a substantial body of research on the relationship between PFAS exposure and metabolites; however, studies that connect these metabolites to both PFAS exposure and biological effect indicators are limited, primarily focusing on changes in blood metabolites. To date, metabolomics research has enhanced our understanding of the comprehensive impact of PFAS exposure on human health and has identified associated metabolites and intermediary markers involved in the onset and progression of various diseases (e.g., liver diseases, glucose and lipid abnormalities, cancer). These metabolites may serve as potential biomarkers for the association between PFAS exposure and biological effects. By synthesizing recent metabolomic studies on PFAS exposure and toxicity, we can elucidate consistent metabolic response patterns to PFAS exposure, revealing common pathways and potential biomarkers affected in both human models and laboratory rodents, as detailed in Table 4.

#### 4.2.1. Blood

Research on the association between PFASs and liver and glucose–lipid abnormalities has identified glycerophospholipids as major associated metabolites with potential as effect biomarkers. Four studies have all found that glycerophospholipids may be the mediating metabolite that mediates the association of PFAS exposure with liver function or dyslipidemia [62,70,88,90]. A mediator analysis of metabolites mediating the association of PFAS exposure with abnormalities in APOB, an index of liver function, showed that glycerophospholipids were the main markers for the association of PFASs with APOB [70]. PFASs were also found to be predominantly associated with glycerophospholipid and amino acid metabolism in a study of the histologic severity of nonalcoholic fatty liver disease, in which differential metabolites were divided into two potential clusters, and cluster 2 was found to be associated with an increased incidence of NASH and higher PFAS concentrations [62]. In dyslipidemia studies, most glycerophospholipids were positively associated with PFASs and were associated with an increased risk of TC abnormalities, and the metabolites that played a mediating role mainly involved glycerophospholipid metabolism, linoleic acid metabolism, and primitive bile acid biosynthesis, in which 24-Hydroxycholesterol, 3alpha,7alpha-dihydroxy-5beta-cholestan-26-al, PC(18:0/0:0), PC(22:5/0:0), GPCho(18:1/18:1), LPC(22:2(13Z,16Z)), LPC(16:0), 9(S)-HODE, 9,10-DHOME, L-glutamate, 4-hydroxybutyric acid, cytosine, PC(14:1(9Z)/18:0), sphinganine, and (S)-beta-aminoisobutyrate were important markers [90]. Similarly, in studies related to plasma cholesterol and triglycerides, a metabolite pattern predominantly involving glycerophospholipids mediated the association between PFASs and triglycerides, showing a negative correlation with triglycerides [88]. In HCC risk studies, high PFOS levels were associated with increased HCC risk, with glucose, butyric acid, α-ketoisovaleric acid, and 7α-hydroxy-3-oxo-4-cholestenoate identified as potential mediators of the association between PFOS exposure and increased HCC risk [97]. The possible mechanism linking PFASs to increased liver cancer risk is through alterations in glucose, amino acid, and bile acid metabolism. In type 2 diabetes (T2D) risk studies, PFASs were found to be positively associated with two metabolite patterns, but these patterns had opposite relationships with T2D risk [91]. The metabolite pattern PC2, predominantly involving glycerophospholipids, was associated with reduced T2D risk, whereas the metabolite pattern PC1, primarily involving diacylglycerols, was associated with increased T2D risk. In studies related to changes in glucose homeostasis, children with high PFAS concentrations were found to have elevated 2 h glucose levels at baseline and during follow-up [93]. Further metabolomic analysis divided differential metabolites into two potential clusters, with the “high-risk” cluster positively correlated with PFASs, mainly involving the metabolism of palmitic acid, hydroperoxylinoleic acid, tyrosine, phenylalanine, arginine, sphingolipids, linoleic acid, and aspartic acid.

In the study of intermediary metabolites associated with PFASs and reproductive development, it was found that PFASs are primarily associated with the metabolism of amino acids and lipids and identified Octanoylcarnitine (C8) and uric acid as potential intermediary biomarkers [76,79,84]. In studies examining maternal PFAS exposure and childhood obesity trajectories before age four, three BMI z-score trajectories in early childhood were identified, in which an increased likelihood of a persistently increasing trajectory type was associated with prenatal PFAS exposure [79]. Further analysis identified octanoylcarnitine (C8) as the sole mediator between PFAS exposure and childhood BMI trajectory. In a study of maternal PFAS exposure associated with gestational age and PTB, PFASs and gestational age and PTB were found to be primarily involved in the pathways of glycerophospholipid metabolism, amino acid metabolism in the urea cycle, and tryptophan metabolism [76]. In another study on PFAS exposure and fetal growth in pregnant women, 10 metabolites, such as glycine, taurine, uric acid, ferulic acid, and the unsaturated fatty acid C18:1, were identified as overlapping with the endpoints of PFASs and fetal growth. Further analysis indicated that uric acid may be a potential intermediate biomarker representing an early response to PFAS exposure and predicting reduced fetal growth [84]. In addition, in studies on prenatal PFAS exposure and childhood liver injury risk, an increase in prenatal PFAS exposure was associated with elevated serum levels of branched-chain amino acids (BCAAs: valine, leucine, and isoleucine), aromatic amino acids (AAAs: tryptophan and phenylalanine), biogenic amine acetylornithine, glycerophospholipid PC aa C36:1, and Lyso-PC a C18:1 in children at high risk for liver injury compared to those at low risk [69].

#### 4.2.2. Liver

Metabolomic studies investigating the association between PFAS exposure and biological effect indicators in the liver primarily focus on animal models, revealing that liver damage and disruptions in glucose homeostasis are primarily attributed to alterations in hepatic lipid metabolism. In a study examining liver lipid metabolism in relation to hepatic steatosis index, significant correlations were observed in males, with eight lipids showing notable associations with HSI [73]. Specifically, PC 36:4, PC 38:5, and TG 56:5 exhibited positive correlations with HSI, while PC 38:6, PE 38:6, TG 52:5, TG 54:7, and TG 56:8 were negatively correlated with HSI. In females, only two lipids demonstrated significant associations, with TG 54:3 positively correlated and TG 52:5 negatively correlated with HSI. A study on the impact of liver metabolites on maternal fasting blood glucose levels identified key metabolic products such as glycerol 3-phosphate and lactosylceramide (d18:1/12:0) that may be associated with changes in fasting blood glucose levels [94]. Additionally, metabolites including 20-Oxo-leukotriene E4, licoagrochalcone B, tobramycin, ajmaline, P,P-Dioctyldiphenylamine, 4-Methyl-2-pentyl-1,3-dioxolane, 3b,17a,21-Trihydroxypregnenone, and TG(20:5(5Z,8Z,11Z,14Z,17Z)/22:6(4Z,7Z,10Z,13Z,16Z,19Z)/o-18:0) were found to be associated with fasting blood glucose levels.

#### 4.2.3. Urine

Population studies utilizing metabolomic analysis of urine revealed eight differential metabolites associated with PFAS exposure and biochemical indicators of kidney function, including urea, creatine, and uric acid [96]. Among these metabolites, farnesyl pyrophosphate and taurine were negatively correlated with PFAS exposure, while S-Inosyl-L-homocysteine, 3α,12α-Dihydroxy-5β-chol-6-en-24-oic acid, 4-hydroxyphenylacetic acid, pyridoxine, versiconal, and cholic acid showed positive correlations with PFAS exposure. Notably, S-Inosyl-L-homocysteine (amino acid class) and farnesyl pyrophosphate (isoprenoid class) exhibited the highest correlation.

## 5. Summary and Outlook

### 5.1. Summary

**Figure 1:**
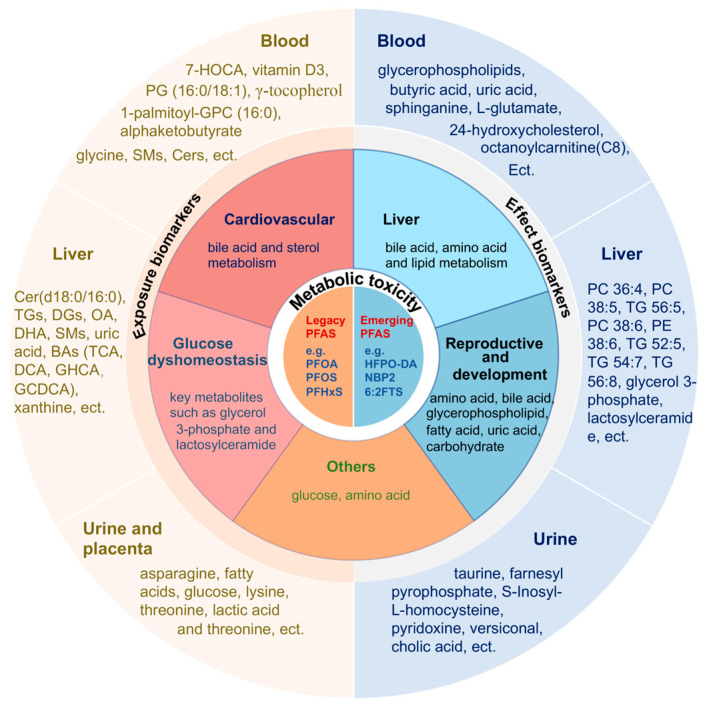
PFAS primarily metabolic toxicity and biomarkers.

Exposure to PFASs has been associated with various biological abnormalities. The advancement of metabolomics technology has provided us with tools to develop an earlier and more comprehensive understanding of the impact of PFAS exposure on disease occurrence and progression mechanisms, as well as facilitating the identification of exposure and effect biomarkers. As illustrated in Figure 1, existing research indicates that PFASs primarily affect liver damage, reproductive toxicity, cardiovascular diseases, abnormal glucose metabolism, kidney diseases, and cancer by influencing the metabolism of amino acids, lipids, and bile acids. Several animal experiments have demonstrated a dose–response relationship of PFASs to alterations in lipid and amino acid metabolites, such as in the metabolome of 0.03 and 0.3 mg/kg PFOS-exposed pregnant rats, where a dose–response relationship was also found in bile secretion, glycine, serine and threonine metabolism, and linoleic acid metabolism, as well as a dose–response relationship in the liver transcriptome in terms of alterations in bile secretion, valine, leucine and isoleucine biosynthesis, and arachidonic acid metabolism [94]. Gao et al. observed an effect of PFOA on lipids, such as unsaturated triglycerides, sphingomyelins, saturated phosphatidylcholines, phospholipid ethers, and other lipids, in a dose–response relationship [108]. In addition, reports of BA metabolism in animal models and in vivo studies have demonstrated that PFAS exposure inhibits de novo synthesis of BA via inhibition of cholesterol 7a-hydroxylase (CYP7A1) and that subsequent inhibition of CYP7A1 leads to the down-regulation of primary BAs (CA, CDCA) [63,71]. Current studies suggest that PFASs primarily induce liver damage by disrupting bile acid, amino acid, and lipid metabolism, reproductive toxicity by perturbing amino acid, lipid, fatty acid, glycerophospholipid, bile acid, uric acid, and carbohydrate metabolism, and lipid and cholesterol abnormalities by disturbing bile acid and cholesterol metabolism. Additionally, PFASs can also influence the occurrence and development of glucose metabolism, kidney diseases, and cancer through metabolic regulation. Although the specific metabolites disrupted by toxicity in different tissues and organs may vary, there are common metabolic disturbances, such as associations with amino acids, lipids, and bile acids observed in almost all abnormalities. Changes in various metabolites in blood, liver, and urine were associated with exposure to PFASs, which offers a reservoir of metabolites that may be associated with PFAS exposure. Some unique metabolites or a combination of multiple metabolites could be potential biomarkers upon further validation. Reviewing the past literature reveals that metabolites associated with PFASs primarily include lipids, amino acids, bile acids, steroids, and acylcarnitines, with changes in blood metabolites dominating the research. Glycerophospholipids show promising potential as effect biomarkers in studies related to PFAS exposure and toxic effects. In addition, we have some limitations with this review in that the alterations in metabolites such as lipids, amino acids, and bile acids identified by this study may not be unique to PFASs alone; however, a comprehensive characterization of the metabolite changes would provide clues to the underlying mechanistic alterations that underlie these adverse outcomes. Further research is needed for a more specific investigation into lipid and amino acid metabolism. Moreover, due to differences in sample matrix and physiological pathological states, different biomarkers may be identified, emphasizing the need for further analysis of specific biomarkers under specific physiological conditions.

### 5.2. Outlook

Current research on PFAS exposure primarily focuses on legacy PFAS compounds, with a predominant emphasis on individual PFAS studies. Some studies have indicated that disturbances in metabolomic profiles caused by PFAS mixtures may be more pronounced than those induced by single PFAS compounds. However, the composite exposure characteristics of perfluoroalkyl substances and their effects on internal metabolites remain unclear. This significantly hampers the accuracy of risk assessments for populations with high exposure to perfluoroalkyl substances. Therefore, there is a need to fill the data gap in metabolomic studies of novel PFAS substitutes and PFAS mixtures. It has also been observed that the toxicity varies among different monomers. Even with higher accumulation levels in the liver, the disruptive effects of their metabolites may not necessarily be greater [85,106]. Studies have highlighted that the novel PFAS substitute NBP2 exhibits developmental toxicity in rats, with oral toxicity slightly lower than PFOS but stronger than HFPO-DA. Comparing the toxicity of different PFAS monomers is crucial for identifying substitutes with lower toxicity. Thus, further research is needed to compare the toxicity of different novel PFAS compounds.

Existing research on the association between PFAS exposure and metabolomics mainly focuses on changes in metabolites associated with PFAS exposure to explore underlying mechanisms. However, there is a lack of research on exposure biomarkers and metabolomic markers that further mediate these effects.

Research on the biological effects of PFASs primarily targets the general population, with limited studies on the effects of exposure on pregnant women, infants, toddlers, and children. Additionally, studies have found higher detection rates among occupational populations near factories, with differential metabolites associated with oxidative stress, impaired fatty acid β-oxidation, and kidney damage. The impact of occupational exposure on worker health cannot be overlooked, highlighting the need for further research on populations with high exposure risks [100].

With the advancement of omics technologies, transcriptomics, metagenomics, and proteomics have become valuable tools for studying the biological effects of PFAS exposure. However, current research lacks comprehensive multi-level investigations combining metabolomics with other omics approaches to elucidate the mechanisms underlying the occurrence and development of PFAS-related effects. There is a need to integrate various omics techniques to provide a more comprehensive understanding of the impact of PFAS exposure and to validate metabolomics findings across multiple levels using other omics methodologies.

## Figures and Tables

**Table 1:** Common PFAS compounds.

Legacy PFAS		
**Name**	**Structural Formula**	**CASRN**
Long-chain PFCA	C*_n_*F2*_n+_*_1_COOH, *n* ≥ 7	-
Long-chain PFSA	C*_n_*F2*_n+_*_1_SO_3_H, *n* ≥ 6	-
**Emerging PFAS**		
**Name**	**Structural Formula**	**CASRN**
Short-chain PFCA	C*_n_*F*_2n+_*_1_COOH, *n* = 2–5	-
Short-chain PFSA	C*_n_*F*_2n+_*_1_SO_3_H, *n* = 2–5	-
HFPO-DA or “GenX”	C_3_F_7_O(CF_3_)CFCOOH	13252-13-6
NBP2	CF_3_CHFOC_3_F_6_OC_2_F_4_SO_3_H	749836-20-2
6:2 FTS	C_8_H_4_F_13_SO_3_H	27619-97-2
6:2 Cl-PFESA	Cl(CF_2_)_6_O(CF_2_)_2_SO_3_^−^	73606-19-6
8:2 Cl-PFESA	Cl(CF_2_)_8_O(CF_2_)_2_SO_3_^−^	83329-89-9
PFOSA	C_8_H_17_SO_2_NH_2_	754-91-6
EtFOSAA	C_8_F_17_SO_2_N(C_3_H_7_)COOH	2991-50-6
MeFOSAA	C_8_F_17_SO_2_N(C_2_H_5_)COOH	2355-31-9
6:2 FTEOs	C_6_F_13_(C_2_H_4_O)*_n_*H, *n* = 4–12	1640092-35-8 ^a^

PFCA, perfluoroalkyl carboxylic acid; PFSA, perfluoroalkane sulfonic acid; HFPO-DA or “GenX”, hexafluoropropylene oxide dimer acid; NBP2, nafion byproduct 2; 6:2 FTS, 6:2 fluorotelomer sulfonic acid; 6:2 Cl-PFESA, 6:2 chlorinated polyfluorinated ether sulfonate; 8:2 Cl-PFESA, 8:2 chlorinated polyfluorinated ether sulfonate; PFOSA, perfluorooctane sulfonamide; EtFOSAA, 2-(N-ethyl-perfluorooctane sulfonamido) acetic acid; MeFOSAA, 2-(N-methyl-perfluorooctane sulfonamido) acetic acid; 6:2 FTEOs, 6:2 fluorotelomer ethoxylates. ^a^ Capstone FS-30, commercial mixture.

**Table 4:** PFAS exposure intermediate metabolites associated with biological effects.

**PFAS**	**Study Object**	**Sample Size**	**Toxic Effect**	**Sample Matrix**	**Main Finding(s)**	**Ref.**
PFHxS, PFHpS, PFOA, PFNA, PFDA, PFOS, PFUdA, 6:2Cl-PFESA, 8:2Cl-PFESA	Residents of Guangzhou, China	278	Liver function indicators (APOB, GGT, DBIL)	serum	78, 58 46 metabolites mediate the association between PFASs and APOB, DBIL, and GGT, respectively The main markers associated with APOB and PFASs were glycerophospholipids; the main marker associated with DBIL was 5-(14-nonadecenyl)-1,3-benzenediol; the main markers associated with GGT were (R)-dihydromaleimide, Ile Leu, (R)-(+)-2-pyrrolidone-5-carboxylic acid, and L-glutamate	Chen Y et al., 2024 [90]
PFOS, PFHxS, PFOA, PFNA	Pregnant African American Newborns in Atlanta, Georgia	267	Premature labor and gestational age at delivery	serum	56 metabolites were associated with both PFASs and birth outcomes Amino acids and protein metabolites were the most abundant, and were associated with PFNA and PFHxS and gestational age and PTB; Carnosine and bile acids were associated with PFASs and gestational age; polyunsaturated omega-6 fatty acids, arachidonic and linoleic acids, and tetradecenoyl levocarnosine were correlated with PFHxS and gestational week; indole-3-acetic acid methyl ester, benzoic acid, and s -lactic acid glutathione were associated with PFASs and premature delivery and gestational age	Taibl KR et al., 2023 [76]
PFHxS, PFOA, PFHpS, PFNA, PFOS, 6:2 Cl-PFESA, PFDA, PFUdA	Male residents of Guangzhou	278	Dyslipidemia (TC and LDL)	plasma	105 and 82 metabolites mediated the association between PFASs and TC and LDL, respectively Most glycerophospholipids were positively associated with PFASs and TC Important markers were 24-Hydroxycholesterol, 3alpha,7alpha-dihydroxy-5beta-cholestan-26-al, PC(18:0/0:0), PC(22:5/0:0), GPCho(18:1/18:1), LysoPC(22:2(13Z,16Z)), LysoPC(16:0), 9(S)-HODE, 9,10-DHOME, L-glutamate, 4-hydroxybutyric acid, cytosine, PC(14:1(9Z)/18:0), sphinganine, and (S)-beta- aminoisobutyrate	Chen Y et al., 2023 [90]
PFOA, PFOS, PFNA, PFUdA, PFDA, PFHxS, PFBS, PFDoA, PFHpA, PFOSA	A birth cohort study in Shanghai, China	1671	Obesity trajectory in children by age 4	plasma	Increased odds of sustained growth trajectories (category 1) among three BMI z-score trajectories were associated with prenatal PFAS exposure Octanoylcarnitine (C8) was the only mediator found in the sum of PFOS, PFNA, PFDA, a total of seven PFASs (PFOA, PFOS, PFNA, PFDA, PFUdA, PFHxS, PFHpA), and BMI trajectory	Zeng X et al., 2023 [79]
23 PFASs, mainly PFHpA, PFOA, PFBS, PFPeS, PFHxS, PFHpS, PFOS, 6:2FTS	Workers and residents in Hubei, China	225	Renal function indicators (Urea, Creatine, Uric acid)	urine	8 differential metabolites were correlated with PFAS and renal function indicators, of which farnesyl pyrophosphate and taurine were negatively correlated with PFASs, and S-Inosyl-L-homocysteine,3α,12α-dihydroxy-5β-chol-6-en-24-oic acid, 4-hydroxyphenylacetic acid, pyridoxine, versiconal, and cholic acid were positively correlated with PFASs S-Inosyl-L-homocysteine and farnesyl pyrophosphate had the highest correlations	He A et al., 2023 [96]
PFHxS, PFOS, PFOA, PFNA	A birth cohort of African American pregnant women	313	Decreased fetal growth	serum	10 metabolites overlapping with PFASs and fetal growth endpoints, including glycine, taurine, uric acid, ferulic acid, 2-hexyl-3-phenyl-2-primary acid, unsaturated fatty acid C18:1, androgen couplers, maternal bile acids, and bile acid-glycine couplers Uric acid is a potential intermediate biomarker of serum PFOA and PFNA associated with reduced fetal growth	Chang CJ et al., 2022 [84]
PFOS, PFHxS, PFOA, PFDA, PFNA, PFUdA	A nested case-control study	50 pairs	Non-viral HCC risk	plasma	High levels of PFOS were associated with increased risk of HCC Four metabolites that were positively associated with both PFOS exposure and HCC risk were glucose, butyric acid, α-ketoisovaleric acid, and 7α-hydroxy-3-oxo-4-cholestenoate.	Goodrich JA et al., 2022 [97]
PFDA, PFNA, PFUdA	A nested case-control study	187	Cholesterol and triglycerides	plasma	50 metabolites were associated with PFASs and triglycerides, and a pattern of metabolites dominated by glycerophospholipids was associated with long-chain PFASs and negatively correlated with triglycerides PFAS-related metabolites not associated with cholesterol	Schillemans T et al., 2023 [88]
PFOS, PFOA, PFNA, PFHxS, PFUdA	The Human Early-Life Exposome project	1105	Risk of liver injury in children	serum	Liver injury risk associated with BCAAs (valine, leucine, and isoleucine), AAAs (tryptophan and phenylalanine), biogenic amine acetyl ornithine, and the glycerophospholipid phospholipids (PCs) aa C36:1 and Lyso-PC a C18:1	Stratakis N et al., 2020 [69]
PFOA, PFOS and PFHxS	Cross-sectional cohort of NAFLD	74	Histologic severity of NAFLD	plasma	Differential metabolites were categorized into two potential clusters, and cluster 2 was associated with an increased incidence of NASH Cluster 2 was positively associated with PFASs and plasma metabolite patterns alters, including elevated ethanolamine phosphate, tyrosine, phenylalanine, aspartate, and creatine, and decreased betaine	Jin R et al., 2020 [62]
PFOA, PFOA, PFHxS, PFDA, PFNA, PFUdA	a case-control study on T2D nested	187 pairs	Type 2 diabetes risk	plasma	PFASs were positively associated with two model metabolites, but two model metabolites were inversely associated with T2D risk 35 differential metabolites such as gamma-butyrobetaine, 3,4,5-trimethoxycinnamic acid, and DHA were associated with T2D, including mainly PC1, which is predominantly diacylglycerol metabolite, and PC2, which is predominantly glycerophospholipid	Schillemans T et al., 2021 [91]
PFOA, PFOS, PFHxS	Hispanic children in Los Angeles	40	glucose homeostasis	plasma	Differential metabolites identified in two clusters, with higher concentrations of PFASs in children categorized as “high-risk” (“cluster 2”) associated with elevated 2 h blood glucose levels The “high risk” cluster was positively associated with PFASs and plasma metabolites related to palmitic acid, hydroperoxylated linoleic acid, tyrosine and phenylalanine and arginine, sphingomyelin and linoleic acid, and aspartic acid metabolism	Alderete TL et al., 2019 [93]
**PFAS**	**Study Object**	**Dose**	**Toxic Effect**	**Sample Matrix**	**Main Finding(s)**	**Ref.**
Eight PFAS mixtures (PFOA, PFNA, PFDA, PFUdA, PFDoDA, PFTrDA, PFTeDA, PFOS),	A/J mice	3 g/mouse/week, 10 weeks	Hepatosomatic index (HSI)	liver	HSI was associated with differential metabolites related to PFAS exposure In males, eight metabolites were correlated with HSI, which was positively correlated with PC 36:4, PC 38:5, and TG 56:5, and negatively correlated with PC 38:6, PE 38:6, TG 52:5, TG 54:7, and TG 56:8; in females, two lipids were significantly correlated, with TG 54:3 and TG 52:5 being positively and negatively correlated with HSI, respectively.	Khan EA et al., 2023 [73]
PFOS	SD rats	0.03 and 0.3 mg/kg, GD1-18d	fasting glucose homeostasis	liver	Key metabolites, such as glycerol 3-phosphate and lactose ceramide may be associated with changes in fasting blood glucose Lactosylceramide (d18:1/12:0), glycerol 3-phosphate and 4-Hydroxy-5-(phenyl)-valeric acid-O-glucuronide, 20-Oxo-leukotriene E4, licoagrochalcone B, tobramycin, ajmaline, 4-Methyl-2-pentyl-1,3-dioxolane, 3b,17a, 21-Trihydroxypregnenone, etc., were strongly correlated with FBG	Yu G et al., 2023 [94]

## Data Availability

No new data were created or analyzed in this study.
